# The relationship between ‘school readiness’ and later persistent absenteeism

**DOI:** 10.1098/rsos.240272

**Published:** 2024-06-26

**Authors:** Megan L. Wood, Lydia Gunning, Mark Mon-Williams

**Affiliations:** ^1^ School of Psychology, University of Leeds, Leeds, LS2 9JT, UK; ^2^ Born in Bradford’s Centre for Applied Education Research, Wolfson Centre for Applied Health Research, Bradford Royal Infirmary, Bradford BD9 6RJ, UK

**Keywords:** school readiness, administrative data, persistent absence, attendance, children

## Abstract

Post-pandemic school absence is an increasing concern for governments worldwide. Absence is associated with poor academic outcomes and long-term illness (physical and mental). Absenteeism increases the risk of financial difficulties in adulthood and involvement in the criminal justice system. We hypothesized that early childhood problems might be an antecedent of absenteeism. We tested this hypothesis by investigating the pre-pandemic association between school readiness and persistent absenteeism using a population-linked dataset. Analyses included 62,598 children aged 5–13 years from the Connected Bradford database (spanning academic years 2012/13 to 2019/20). Special educational needs status, English as an Additional Language status, socioeconomic status, sex and ethnicity were covariates significantly associated with persistent absenteeism. Children who were not ‘school ready’ had increased odds of being persistently absent later in their education journey after controlling for these covariates. School readiness was associated with even greater odds of being persistently absent over two or more years. These findings show (i) the seeds of absenteeism are sown early in childhood; (ii) absenteeism shows the hallmark of structural inequities; and (iii) the potential of ‘school readiness’ measures to identify children at risk of long-term disengagement from the education system.

## 1. Introduction

Good school attendance is key for both academic and later life success, but schools across the world—including the UK, the USA, Australia and Canada—are facing an attendance crisis following the pandemic [1–5]. The devastating disruption and impact on pupils’ mental and physical health have resulted in a situation where teachers suggest that many children ‘never got back into the habit of regularly attending school’ [6].

In the UK, absenteeism is a major concern for school leaders and policymakers, with data from the 2022/2023 autumn term showing that nearly a quarter of pupils missed at least 39 half-day sessions, which according to the UK’s Department for Education (DfE) means they are considered a ‘persistent absentee’ (attendance below 90%). The number of children classified as being ‘persistently absent’ has risen over time, particularly between 2017–2018 and 2020–2021 with the more recent figures attributed to the impact of the COVID-19 pandemic. These figures, which show no sign of plateauing, led to a formal inquiry by the UK Government’s Education Committee in September 2023 [7].

The high rates of absenteeism are alarming for governments because of the well-documented, long-term negative consequences. Absenteeism limits students’ opportunities for learning and creates additional and widespread consequences, both immediate and long term [8]. For example, students who are more frequently absent perform more poorly academically [9–13]. A prospective sample of American children followed from birth through high school showed poor assessment scores across a range of subjects at age 15 years after absenteeism in the first year of formal education [14]. Furthermore, the UK’s Department for Education reported that higher levels of absenteeism across Key Stage 2 (KS2; 7–11 years of age) and Key Stage 4 (KS4; 14–16 years of age) were associated with lower attainment [15]. Eighty three per cent (83.9%) of students who attended all sessions over KS2 achieved the expected standard compared to 40.2% of students who were persistently absent, while 83.7% of students in KS4 passed their English and Mathematics examinations (General Certificate of Education) compared to 35.6% of students who were persistently absent.

Absenteeism can also lead to suboptimal social behaviour and cause individuals to feel more alienated from their peers [12,16]. The impact of absenteeism extends far beyond childhood and adolescence, however, with research demonstrating that absenteeism is associated with economic difficulties in adulthood [14] and poorer mental health [17–19]. Furthermore, students performing less well academically owing to absenteeism are at increased risk of myriad adverse health outcomes. For example, pupils missing a large proportion of school are more likely to exhibit risky health behaviours such as smoking, drug and alcohol abuse [20–22], which, in some cases, leads to involvement with the criminal justice system [23]. Absenteeism has been found to be an important predictor of poor lifetime health in general [24]. This includes a predisposition to develop potentially preventable chronic conditions such as heart conditions and diabetes owing to lower education levels [25,26].

There is therefore an urgent need to understand the factors that lead to absenteeism and determine how children at risk of absenteeism can be identified before they disengage from the school system. Early intervention and support could be put in place for the child and their family and help mitigate the impact of absenteeism if we were able to identify the risk earlier. We hypothesized that early childhood factors might be contributing to the downstream problems that cause disengagement with the education system. Many countries conduct holistic ‘school readiness’ evaluations where children are assessed on both academic (e.g. numeracy) and non-academic abilities (e.g. social skills), including Canada, Australia and the USA [27–31]. In England, the Early Years Foundation Stage Profile (EYFSP) is a statutory assessment conducted by teachers to indicate whether a child is considered ‘school ready’ at school entry. Previous research suggests that such assessments can indicate later ‘vulnerability’. For example, school readiness assessments have been used to identify children at risk of autism [32] and special educational needs (SEN) more generally [27,33].

We therefore tested the relationship between England’s statutory teacher-reported ‘school readiness’ assessment and children later becoming ‘persistently absent’. We also investigated the role of student demographics (e.g. ethnicity, sex, SEN status) in this relationship. A focus was placed on ‘pre-pandemic’ data, to begin to identify the patterns and associations between early childhood problems and absenteeism before this was further complicated by factors attributable to the pandemic (e.g. school closures, etc.).

## 2. Material and methods

### 2.1. Participants and study setting

Data were collated from Connected Bradford, a linked database for over 8 00 000 citizens across the Bradford district, UK [34]. DfE records were linked across multiple time points, spanning academic years 2012–2013 to 2018–2019 (i.e. prior to the COVID-19 pandemic). Data were extracted in November 2022. School absence records were obtained for children aged 5–13 years. Within the Connected Bradford database, all individuals are assigned a unique personal identifier, which is the same across all datasets, regardless of its origin (i.e. education, healthcare, etc.). This personal identifier is used to link across all datasets and produce a single ‘research-ready’ cohort for analysis. To be included in analyses, individuals were required to have complete data for all variables (see §2.2.4). The final sample contained 62 598 children (see table 1 and electronic supplementary material, file S1) with approximately 11% of the original cohort excluded owing to missing ethnicity data.

**Table 1.** Demographics of the analytic sample as a function of persistent absenteeism.

	Persistent absentee		Total (*n* = 62598)
	Yes (*n* = 5671)	No (*n* = 56927)	
School ready	1862 (32.8%)	35 763 (62.8%)	37 625 (60.1%)
Has English as an Additional Language	2279 (40.2%)	20 091 (35.3%)	22 370 (35.7%)
Receives support for special educational needs	1891 (33.3%)	8912 (15.7%)	10 803 (17.3%)
Eligible for free school meals	3227 (56.9%)	14 033 (24.7%)	17 260 (27.6%)
Female	2644 (46.6%)	28 445 (50.0%)	31 089 (49.7%)
Ethnicity			
White British	2702 (47.6%)	29 677 (52.1%)	32 379 (51.7%)
Pakistani	1584 (27.9%)	17 137 (30.1%)	18 721 (29.9%)
Other	1385 (24.4%)	10 113 (17.8%)	11 498 (18.4%)

### 2.2. Variables

#### 2.2.1. School readiness

Various school readiness assessments are conducted globally. In England, a ‘good level of development’ is a binary variable derived from the EYFSP, which is used to determine whether a child is considered ‘school ready’. Teachers use a three-point scale (emerging, expected, exceeding) to rate a child’s performance on seven domains of development. If a child’s teacher determines a child is performing at either expected or exceeding levels across five early learning domains (‘physical development’, ‘communication and language’, ‘personal, social and emotional development’, ‘mathematics’ and ‘literacy’), they are said to have reached a good level of development and are thus school ready [35]. Children rated as ‘emerging’ in any of the early learning goals are defined as not being school ready. In 2013, changes were made to the EYFS framework. We therefore focused our analyses on the post-2013 EYFSP to ensure the data were comparable (see Wood *et al*. for further details [33]).

#### 2.2.2. Absenteeism

Children were identified as being a ‘persistent absentee’ if they were recorded as having 90% attendance or less over the course of at least one academic year. This is regardless of the reason for the absence or whether the absence was authorized or not. Persistent absenteeism is a binary variable (‘yes’/‘no’), which was readily available in the dataset obtained from the DfE. Meanwhile, children were identified as a ‘recurrently persistent absentee’ if they were identified as a persistent absentee for two or more academic years (at any point throughout their education). These terms were used to differentiate between children who had a substantial number of absences within a single year only (perhaps owing to a single bout of illness) and those who consistently missed a substantial amount of school. Analyses exploring the association between school readiness and being a recurrently persistent absentee are reported in electronic supplementary material, file S2. Absence data were collected on an academic year basis. There were therefore multiple absence records for each individual. A child was identified as a persistent absentee if, across their academic attendance records, they were recorded as a persistent absentee in at least one of those academic years.

#### 2.2.3. Additional covariates

Additional covariates were included according to previous similar research [27,33]. Ethnicity was obtained from census records (provided by the DfE). Owing to the distribution of ethnic groups, children were re-categorized as ‘White British’, ‘Pakistani’, ‘Other’. Children whose ethnicity was recorded as ‘Unknown’ or was missing were excluded from analyses. Children were recorded as having SEN if their records *ever* indicated the child had a School Action or Early Years Action Plan, a School Action Plus or Early Years Action Plus Plan, received SEN support, had an SEN statement or had an Education, Health, and Care Plan (up to the 2018/2019 academic year). Note that SEN indicates children who have been identified as having SEN and are likely to be receiving some additional support, rather than children who need support but have not yet been formally identified. Any children who were not recorded as having any of the above plans or support were recorded as not having SEN. Eligibility for free school meals (at any time) was used as a proxy measure for socioeconomic position (see https://www.gov.uk/apply-free-school-meals for further detail). Sex at birth (male, female) was obtained from census records. Lastly, children with English as an Additional Language (EAL) were identified using the census records.

#### 2.2.4. Statistical analysis

Several logistic regression models were conducted to understand the relationship between school readiness and absenteeism. In line with similar studies that explored the utility of school readiness to identify later risk, we report odds ratios (ORs) to demonstrate the effect of these relationships [27,32,33]. Logistic regression analyses were conducted owing to the binomial nature of the variables of interest [36]. The first set of analyses sought to determine whether failing to be school ready is associated with an increased risk of becoming a persistent absentee at any stage of a child’s academic career. First, an unadjusted baseline logistic regression model with school readiness (yes, no) as the predictor and persistent absentee status (yes, no) as the outcome was performed. Second, a multilevel logistic regression model includes school readiness, persistent absentee status, covariates (EAL, eligibility for free school meals, SEN status, sex and ethnicity), plus the inclusion of school ID as a random intercept to account for clustering across schools. Multilevel models are particularly useful for accounting for both individual-level and contextual effects [37]. Lastly, a multilevel logistic regression model included the additional predictors where the reference category is indicated by an asterisk (*): ethnicity (White British*, Pakistani, Other), eligibility for free school meals (yes, no*), sex (male, female*), EAL (yes, no) and SEN status (yes, no). All analyses were conducted using R [38] (v. 4.0.2 via a Jupyter notebook hosted on the Google Cloud Platform). Additional analyses were conducted to explore the association between school readiness and recurrently persistent absences (reported in electronic supplementary material, file S2).

## 3. Results

The frequencies and percentages of children identified as a persistent absentee at any point in their academic career depending on whether they were school ready are displayed in table 2.

**Table 2.** The number of pupils identified as a persistent absentee as a function of school readiness (school ready/not school ready).

	Persistent absentee	Not a persistent absentee	Total
School ready	1862 (32.8%)	35 763 (62.8%)	37 625
Not school ready	3809 (67.2%)	21 164 (37.2%)	24 973
**Total**	5671	56 927	62 598

**Figure 1.**
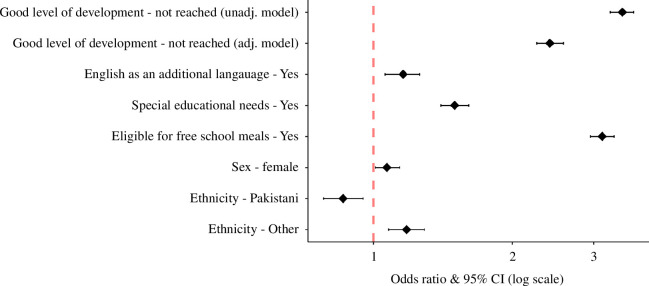
Odds ratios and 95% confidence intervals for the association between school readiness and persistent absentee status (unadjusted model) and school readiness and persistent absentee status + covariates (adjusted model).

The outcomes for the multilevel multinomial logistic regressions are presented in figure 1.

The odds of children becoming a persistent absentee at any point in their academic careers were significantly higher for those who were not school ready than for those who were school ready. This was observed for both the unadjusted model (model 1: OR 3.46, 95% confidence intervals (95% CI) 3.26–3.66, *p *< 0.001) and the model controlling for covariates (model 2: OR 2.41, 95% CI 2.26–2.58, *p *< 0.001).

In model 2, several of the covariates were also significantly associated with persistent absenteeism. The odds of children becoming a persistent absentee were higher for children with EAL (OR 1.16, 95% CI 1.06–1.26, *p* = .001). This was also the case for children who had SEN, compared to those who did not (OR 1.50, 95% CI 1.40–1.61, *p *< 0.001); children eligible for free school meals, compared to those who were not eligible (OR 3.13, 95% CI 2.95–3.32, *p *< 0.001), and for females, compared to males (OR 1.07, 95% CI 1.01–1.14, *p* = 0.021). In order to ensure missingness was not a factor, model 2 was run a second time but included approximately 11% of children who had missing ethnicity data. The results of the analyses with and without the ‘missingness’ data were comparable (OR 2.41 without and OR 2.35 with).

Pakistani heritage children had decreased odds of becoming a persistent absentee compared to White British children (OR 0.86, 95% CI 0.78–0.95, *p* = 0.003), while children whose ethnicity was recorded as ‘Other’ had higher odds than White British children (OR 1.18, 95% CI 1.08–1.29, *p *< .001).

Analyses also revealed that not being school ready was associated with even greater odds of being a recurrently persistent absentee (adjusted model; OR 2.91, 95% CI 2.58–3.28) than being persistently absent at any point (see electronic supplementary material, file S2).

## 4. Discussion

We sought to test the hypothesis that school absenteeism is related to factors occurring before the point of school entry. We reasoned that if we were unable to reject this hypothesis, then it would suggest that measures of school readiness might be useful for governments wishing to identify children ‘at risk’ of absenteeism at an early stage in their educational journey. We tested the hypothesis using England’s statutory teacher-reported assessment of school readiness (the EYFSP). We found that several covariates were associated with absenteeism (ethnicity, socioeconomic circumstances, SEN status, sex and EAL status). We found that children who were not school ready were at increased risk of becoming a persistent absentee at some point across their school career even after controlling for these covariates. This was also the case when considering recurrently persistent absentees who had been persistently absent for two or more academic years.

The findings corroborate previous research showing the usefulness of assessments of school readiness in the early identification of children who may need additional support [27,33]. This is an important finding for school leaders, policymakers and families, as it suggests that children at increased risk can be identified prior to attendance problems arising.

Potential explanations for this relationship could be related to the engagement levels of parents, with those who are more engaged better preparing their children for the learning environment [39], resulting in children who are more excited to learn. Such parental engagement may continue throughout the child’s school journey, in the form of helping with homework and ensuring children arrive at school ready to learn (e.g. with better nutrition, sleeping patterns, etc.). Alternatively, not being school ready may be indicative of underlying needs, such as neurodivergence or mental health issues that are not currently being met, thus making school difficult for these children [27,33].

We found socioeconomic circumstances (as measured by eligibility for free school meals) were a major risk factor for persistent absence in addition to school readiness. Previous research has consistently found that children from lower socioeconomic backgrounds are more likely to be absent from school [40,41]. Ethnicity was also found to be associated with persistent absenteeism, with children of Pakistani heritage having significantly lower odds of becoming a persistent absentee compared to children of White British heritage. This aligns with previous research in the USA, suggesting that children from Asian, Mexican Hispanic and Black backgrounds were less likely to be persistent absentees compared to White Americans [40].

While the present findings provide insight into how data might be used to identify children at risk of absenteeism, the issues around *why* children become disengaged from the school environment (leading to increased absence) are complex and multifaceted [42,43]. For example, qualitative work has highlighted a wide array of reasons why pupils may not attend school every day, such as the cost-of-living crisis and poverty issues, the mental health epidemic affecting young people, lack of support (particularly for neurodivergent pupils) and the breakdown of the partnership between the school and families [44,45]. Moreover, many children highlighted the lack of a sense of belonging to school as a key barrier to attending and feeling unsupported as they navigate key transitional periods [46]. Thus, much more work and investigation are required to begin to re-engage pupils and their families with the education system, particularly after the COVID-19 pandemic [45]. Corcoran and colleagues have highlighted that absenteeism as a result of mental health issues (often known as ‘emotionally based school non-attendance’) is quite often driven by a poor home–school relationship [47]. Therefore, one avenue to explore in improving attendance might involve working more closely with the families of pupils who begin their formal education not school ready to ensure the whole family feels supported.

### 4.1. Limitations and future work

It is important to note that the data for this study were obtained from a single district in West Yorkshire, UK, and thus act as a ‘proof of concept’ for the potential of using administrative data to identify risk. Additional work should analyse records from other regions across the UK. These findings raise further questions about which early interventions or support may be most appropriate, and we intend to investigate these questions in subsequent studies. The nature of the data used also introduced limitations. Administrative data allow a large analytical dataset with minimal time or resource implications (in comparison to data obtained from experimental studies) but can introduce issues related to missingness. In the current study, individuals with missing gender data accounted for <0.1% of the total sample, so they were unlikely to skew the sample. On the other hand, 11.6% of the sample had missing ethnicity data. In order to increase confidence that missingness did not exert an undue influence, we ran the analyses with and without the children who had missing ethnicity information. The results of these analyses were comparable, so we are confident that the results are robust to missingness. However, missingness is almost invariable in studies involving administrative data and thus a limitation of a study of this type—a limitation that can hopefully be addressed through analysis of comparable datasets in other parts of the country.

## 5. Conclusion

This study highlights that school readiness assessments can identify pupils at increased risk of subsequent poor school attendance. Such assessments could be used to identify needs early so that targeted interventions and support can be put in place in a timely fashion. This research adds to a body of work that unequivocally demonstrates children who are not ‘school ready’ may be at risk of multiple vulnerabilities related to health, education and development. We investigated the relationship between absenteeism and school attendance using pre-pandemic data so that we could avoid all the additional complications associated with school attendance following the impact of COVID-19. The fact that countless children had disrupted pre-school experiences before the pandemic leads us to assume that increasing numbers of children are likely to be entering school without the appropriate level of ‘readiness’. Our findings suggest that this, in turn, will create an epidemic of absenteeism in future years. Furthermore, pandemic-related ‘school hesitancy’ is becoming ever more apparent in the current cohort of school children, leading to the possibility that this absenteeism crisis could worsen as these pupils work their way through the educational system. Our results strongly suggest that urgent remedial action is required in the early years of schooling to avert this societal crisis from becoming entrenched over the forthcoming decade.

## Data Availability

All data are from a pseudonymized population-linked database hosted within a Secure Data Environment (SDE) and cannot therefore be shared with the manuscript. Interested parties may submit an Expression of Interest to the Connected Bradford Executive Board for data access by contacting Cbradford@bthft.nhs.uk. All code used for analyses are included in supplementary materials [48].
